# Neutrophil extracellular traps have active DNAzymes that promote bactericidal activity

**DOI:** 10.1093/nar/gkae1262

**Published:** 2024-12-31

**Authors:** Ti-Hsuan Ku, Nikhil Ram-Mohan, Elizabeth J Zudock, Ryuichiro Abe, Samuel Yang

**Affiliations:** Department of Emergency Medicine, Stanford University, 240 Pasteur Drive Rm 0300 Stanford, CA 94305, USA; Department of Emergency Medicine, Stanford University, 240 Pasteur Drive Rm 0300 Stanford, CA 94305, USA; Department of Emergency Medicine, Stanford University, 240 Pasteur Drive Rm 0300 Stanford, CA 94305, USA; Department of Emergency Medicine, Stanford University, 240 Pasteur Drive Rm 0300 Stanford, CA 94305, USA; Department of Emergency Medicine, Stanford University, 240 Pasteur Drive Rm 0300 Stanford, CA 94305, USA

## Abstract

The mechanisms of bacterial killing by neutrophil extracellular traps (NETs) are unclear. DNA, the largest component of NETs was believed to merely be a scaffold with antimicrobial activity only through the charge of the backbone. Here, we demonstrate for the first time that NETs DNA is beyond a mere scaffold to trap bacteria and it produces hydroxyl free radicals through the spatially concentrated G-quadruplex/hemin DNAzyme complexes, driving bactericidal effects. Immunofluorescence staining showed potential colocalization of G-quadruplex and hemin in extruded NETs DNA, and Amplex UltraRed assay portrayed its peroxidase activity. Proximity labeling of bacteria revealed localized concentration of radicals resulting from NETs bacterial trapping. *Ex vivo* bactericidal assays revealed that G-quadruplex/hemin DNAzyme is the primary driver of bactericidal activity in NETs. NETs are DNAzymes that may have important biological consequences.

## Graphical Abstract

**Graphical Abstract:**
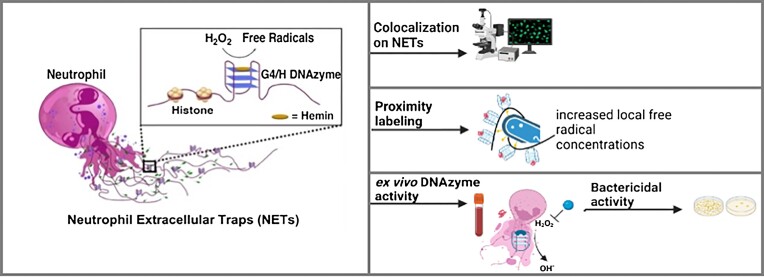

## Introduction

Neutrophils represent a substantial component of the innate immune system, serving as the primary defense against infecting pathogens. Their ability to eliminate pathogens is carried out through various mechanisms, such as phagocytosis, degranulation and the synthesis of neutrophil extracellular traps (NETs), which were discovered in 2004 (1). NETs are intricate networks composed of decondensed chromatin DNA fibers that ensnare and immobilize invading pathogens, effectively impeding their dissemination. Upon entrapment, the immobilized pathogens become exposed to potent and often lethal concentrations of histones, azurophilic granules, specific granules, tertiary granules and cytosolic proteins intricately bound to the DNA fibers released during the process of NETosis. However, depending on the pathogen, like *Pseudomonas aeruginosa* and *Staphylococcus aureus* (SA), NETs may have an incomplete killing effect and eventually aid with killing through the complement (2,3). Additionally, conditions inducing NETosis also affect the NETs bactericidal properties (2). Although NETs play a significant role in eliminating pathogens, they can also contribute to various immunopathologies, such as delayed tissue repair, inflammation, vaso-occlusion and autoantibody generation (4–11). Even though the individual constituents of NETs are known to be involved in both pathogen clearance and the development of immunopathologies, the overall mechanism by which NETs operate remains incompletely understood. Despite being considered a mere scaffold for associated proteins and pathogen sequestration, DNA, the most prevalent component of NETs, has recently been found to play a more active role. This is evidenced by the observation that DNase treatment leads to a 2-fold increase in bacteremia, abolishment of NETs-mediated cytotoxicity *in vitro*, reduction in levels of citrullinated histones associated with NETs and direct disruption of bacterial cell membrane through cation chelation (12).

DNA has the capacity to adopt diverse secondary structures beyond the conventional B-form, including the i-motifs, triple helices and G-quadruplexes (G4s). Through Hoogsteen hydrogen bonding, four guanine bases can come together to create a square planar structure known as a guanine tetrad (G-tetrad or G-quartet), and the stacking of two or more G-tetrads results in the formation of a G4 structure. The utilization of genome-wide high-resolution sequencing revealed the presence of over 700,000 unique G4 structures within the human genome, with a notable enrichment observed in promoter regions surrounding transcription start sites (13–15). Recent physiological findings have established a noteworthy interplay between G4 structures and hemin within living cells, wherein G4 structures exhibit a remarkable capacity to sequester free hemin, thereby serving as a protective mechanism against hemin-induced oxidative damage (16–18). The G4/hemin (G4/H) complex has been recognized for its ability to emulate the functionality of a peroxidase, facilitating the breakdown of hydrogen peroxide (H_2_O_2_) to generate hydroxyl (OH•) free radicals (FRs) (19). This process can occur directly or via superoxide intermediates, ultimately driving the Haber–Weiss reaction (20,21). Many chemically diverse small molecules like BRACO19, TMPyP4 and hemin analog N-methyl mesoporphyrin IX (NMM) that are highly specific to G4s have been widely used to block G4 and inhibit DNAzyme activity without affecting hemin-dependent protein function (22–27). While the DNAzyme activity of the G4/H complex has been well-established through various *in vitro* studies, and the presence of G4 structures in the genome and their role in sequestering hemin have been demonstrated *in vivo*, it remains unclear whether G4 structures exist in the extruded DNA of NETs, their capacity to form the DNAzyme with hemin and generate OH• and their contribution to bactericidal activity in NETs.

## Materials and methods

### Isolation of human blood neutrophils

Human neutrophils were isolated from a healthy donor after informed consent, collected under protocol (70759) approved by Stanford University’s Institutional Review Board, using the EasySep Direct Human Neutrophil Isolation Kit, following the protocol provided by the vendor (#19666, StemCell Tech, Vancouver, BC, Canada). The isolated neutrophils were then resuspended in Dulbecco's Phosphate-Buffered Saline (DPBS, #14190–144, Gibco, Thermo Fisher Scientific) without calcium and magnesium, supplemented with 1 mM ethylenediaminetetraacetic acid (#AAJ15694AE, Thermo Fisher Scientific).

### *In vitro* neutrophil NETosis stimulation, NETs isolation and quantification

The freshly isolated neutrophils were immediately seeded in 12-well plates at a density of 1 × 10^6^ cells per well. The cells were incubated at 37°C for 30 min and then stimulated with 100 nM phorbol 12-myristate 13-acetate (PMA) (#400145, Cayman Chemical) in phenol-red free RPMI-1640 (#11835030, Gibco, Thermo Fisher Scientific) supplemented with 2 mM calcium chloride (#21115, MilliporeSigma) at 37°C in the presence of 5% CO_2_. After 4 h, each well was carefully washed twice with 1 ml of DPBS and then treated with 500 μl of DPBS containing 10U Alu I restriction enzyme (#ER0011, Thermo Fisher Scientific) for 30 min at 37°C. The supernatant from each well was collected and centrifuged for 5 min at 300 × *g* at 4°C to remove cells and cell debris. The supernatants rich in NETs were then further purified using the Monarch genomic DNA purification kit which contains Proteinase K and RNase A to degrade NETs-associated proteins and RNAs, respectively (#T3010L, New England Biolabs). Purified NETs DNA was eluted using sterilized, nuclease-free water (#AM9930, Thermo Fisher Scientific) and subsequently heated to 95°C for 5 min to denature any residual proteins. Following this, the NETs DNA was subjected to centrifugation at 300 × *g* for 1 min. The supernatant was then collected, allowed to cool to room temperature and incubated for 60 min. NETs DNA were quantified using the Quant-iT PicoGreen dsDNA Kit (#P11496, Molecular Probes, Thermo Fisher Scientific), following the manufacturer’s instructions, and assessed for quality by measuring the A260/280 ratio and stored at 4°C for use.

### Immunofluorescence staining for NETs visualization

We modified Biffi *et al.* (28) human cell G4s immunofluorescence staining method for NETs G4 visualization. Briefly, we isolated human neutrophils from a healthy donor. The freshly isolated neutrophils (5 × 10^5^) were immediately seeded in four-well chamber slides (#154526, Thermo Fisher Scientific) with 1 ml phenol-red free RPMI-1640 supplemented with 2 mM calcium chloride (#21115, MilliporeSigma). The chamber slides were incubated in a CO_2_ incubator under 37°C for 20 min. The neutrophils were subjected to treatment with or without *Escherichia coli* ATCC 11775 for a duration of 2 h to initiate NETosis and generate NETs. The generated NETs were rinsed twice with cold DPBS and then fixed with 4% paraformaldehyde for 30 min at room temperature. Subsequently, the NETs were washed three times with DPBS and permeabilized with 0.1% Triton X-100 in DPBS for 1 h at 4°C. Afterward, the NETs were washed three more times with DPBS and treated with 50 μg/ml RNase A (#T3018, New England Biolabs) for 1 h at 37°C. Following this, the NETs were blocked with a mixture of 1% skim milk and 0.1% Tween-20 in DPBS at 4°C overnight. Immunofluorescence stain was performed using standard methods with or without BG4 (#MABE917, MilliporeSigma), anti-FLAG (#F1804, MilliporeSigma) and anti-mouse IgG Alexa 594 (#AB150116, abcam) antibodies for G4 detection; 1D3 (#Ab00982-10.0, absolute antibody) and anti-human IgG Alexa-488 (#A-11013, Thermo Fisher Scientific) antibodies for hemin; anti-Cit-Histone (ab5103, abcam); and Anti-myeloperoxidase antibody (#ab208670, abcam) and anti-rabbit IgG Alexa 647 (#ab150083, abcam) antibodies for citrullinated-histone and myeloperoxidase, respectively. NETs DNA was stained with Hoechst 33342 (#62249, Thermo Fisher Scientific). Digital images were recorded using a Nikon ECLIPSE Ti2-E microscope and analyzed with ImageJ software. Colocalization between G4 and hemin was estimated from the images using the JACoP plugin (29) for ImageJ.

### Comparison of fluorescent area between intact neutrophils versus NETs

For each experimental condition, between eight and nine random fields of view were analyzed. Image analysis was conducted using FIJI software (ImageJ, NIH, USA). Each field of view was processed to enhance contrast and remove background noise, followed by automated counting of intact cells and fluorescent areas. Manual adjustments were made as necessary to ensure accuracy in cell counting and the number of intact cells per field of view was recorded. For NETs, % of fluorescent area per field of view was recorded. Statistical analyses were performed using GraphPad Prism 9. Differences between groups were analyzed using an unpaired *T*-test.

### *In vitro* bactericidal assay

Monoclonal bacteria, *Enterobacter cloacae* (EC), were cultured in LB medium and shaken at 37°C overnight; a volume of 100 μl of cell suspension was introduced into 3.9 ml of fresh LB medium and cultured for an additional 3–4 h prior to use. The bacterial cells were collected by centrifugation and then resuspended in a 10 mM KCl buffer (#60142, MilliporeSigma) to achieve an optical density of 1.0 at 600 nm (OD 600). Subsequently, the bacteria were diluted to 0.03 OD 600 using a sterile KCl buffer or equivalent to 2 × 10^6^ colony-forming unit per ml. Fifty microliter bacterial solution was then combined with various combinations of 300 ng NETs DNA, 300 ng Telo G4, 10 μM hemin, 5 mM H_2_O_2_ and KCl buffer to a total volume of 1 ml and incubated at 37°C for 30 min. Afterward, the solution was diluted 10 times, and an aliquot of 50 μl of the bacterial suspension was spread onto an NB agar plate for overnight growth in a 37°C incubator. The bacterial colonies on the plates were then counted. Synthetic Telo G4 sequence: TTAGGG TTAGGG TTAGGG TTAGGG TTAGGG TTAGGG TTAGGG TTAGGG TT (Integrated DNA Technologies).

### NETs DNA fragmentation and electrophoresis

Three hundred nanogram of purified NETs DNA was subjected to distinct enzymatic treatment using 20 units EcoR I (#R0101S, New England Biolabs) or 4 units DNase I (#M0303L, New England Biolabs) for 60 min at 37°C, respectively. Subsequently, both enzymatic reactions were terminated through heat inactivation at 75°C for 10 min. The resulting NETs DNA samples were subjected to electrophoresis on a 1.3% agarose gel, followed by visualization using ethidium bromide staining.

### NETs DNAzyme peroxidase assay

In this study, we investigated the peroxidase capability of the G4/H DNAzyme within purified NETs DNA induced by PMA. Specifically, we assessed its capacity to oxidize Amplex UltraRed (AR) reagent (#A36006, Thermo Fisher Scientific) in the presence of H_2_O_2_. (#A22188, Component E, Thermo Fisher Scientific) A standard curve of horseradish peroxidase (HRP) (#A22188, Component D, Thermo Fisher Scientific) activity on AR was initially established to serve as a reference. Subsequently, we determined the optimal quantity of Telo G4 (300 ng, equals 2 mU of HRP) required to achieve comparable enzymatic activity. The same mass of NETs DNA was employed as Telo G4. Four units DNase I, 20 units EcoR I, 150 μM G4-specific competitive inhibitor BRACO19 (#SML0560, MilliporeSigma), the G4-binding hemin analog 150 μM NMM (#396879, Santa Cruz Biotechnology) and 2 mM FR scavenger vitamin C (#11140, MilliporeSigma) were tested to abrogate the DNAzyme activity.

### NETs DNA proximity labeling assay

Single colonies of EC and *S. aureus* ATCC 29213 (SA) were cultured in the LB medium and shaken at 37°C overnight before usage. The bacterial cells were collected by centrifuging, DPBS washing 5 times and redispersed in 10 ml KCl buffer with an optical density of 1.0 at 600 nm (OD 600). One milliliter bacterial solution was mixed with different combinations of 20 mU of HRP, 6.67 µg NETs DNA, 3 µg Telo G4, 10 μM hemin, 10 mM H_2_O_2_ and 10 mM Biotin-tyramide (#SML2135, MilliporeSigma) reagent incubating at 25°C for 45 min. After being washed five times with DPBS, the sample was incubated with FITC Conjugated Avidin (10 µg/ml) (#21221, Thermo Fisher Scientific) for a duration of 2 h. Following this incubation, the sample is washed five more times with DPBS before measuring the fluorescence signals using a fluorescent plate reader (BioTek Synergy LX, Agilent).

### *Ex vivo* bactericidal assay

Neutrophils were collected using the previously described method. The freshly isolated neutrophils were promptly seeded into 12-well plates at a density of 2 × 10^6^ cells per well in 500 μl DPBS containing 10 ng interleukin 8 (IL-8) and 2 mM CaCl_2_ and then agitated at 37°C for 20 min. Subsequently, 10 μl of heat-inactivated human serum with cytochalasin D (10 μl/ml) (#C8273, MilliporeSigma) was added to each well, followed by shaking and incubation for 15 min. Next, the samples were treated with 100 U/ml of RNase for 10 min. To demonstrate the significance of NETs DNAzyme in extracellular killing, 50 μM BRACO19, 50 μM NMM, 10U DNase I and 2 mM vitamin C were pre-treated 5 min prior to bacterial addition (5 × 10^3^ bacteria/well). The samples were then agitated and incubated for 40 min. Afterward, each sample was cultured on Nutrient Agar Plate overnight for colony counting.

## Results

### Co-localization of G4 and hemin in NETs

**Figure 1.**
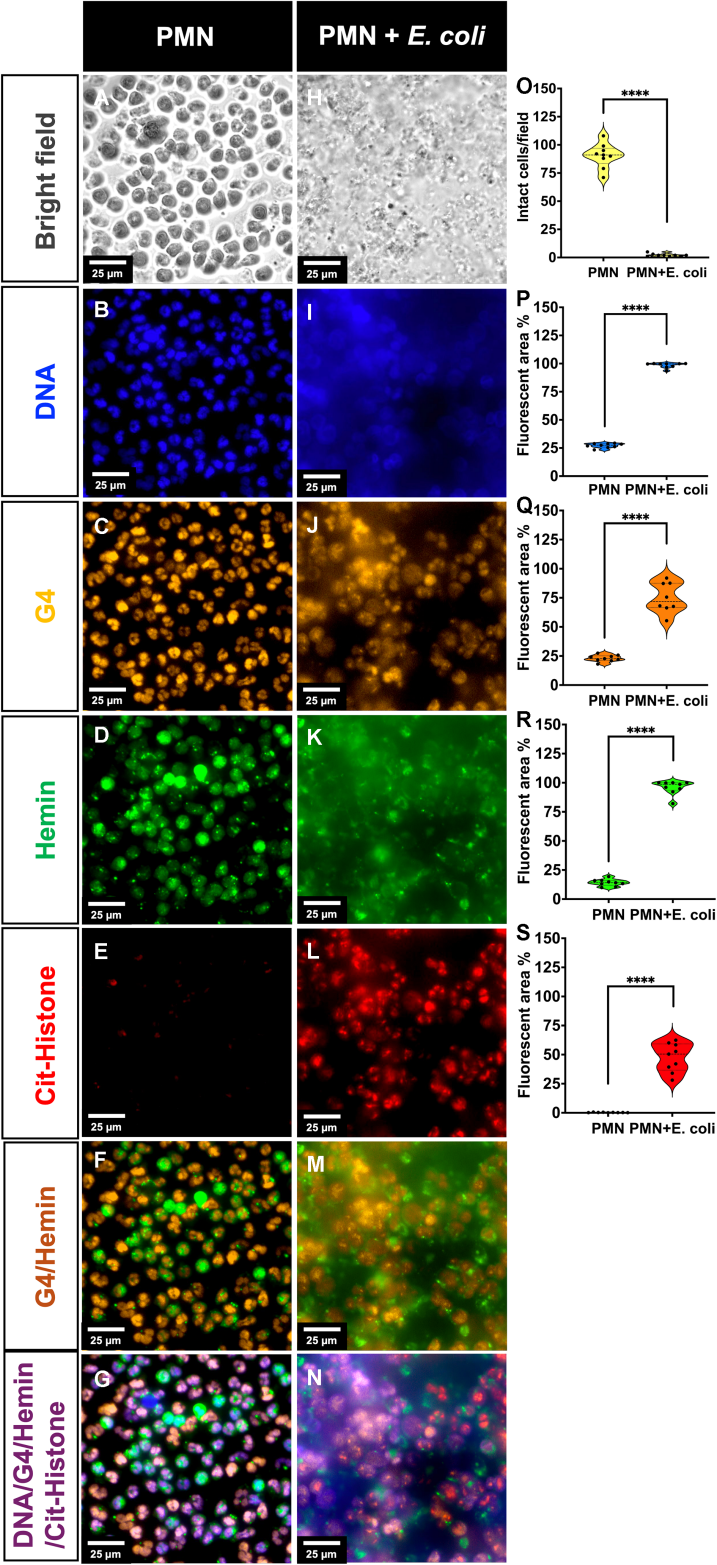
Immunofluorescence stain of putative G4/H colocalization on NETs. (**A**–**G**) Unstimulated neutrophils (PMN). (**A**) Bright field. (**B**) Condensed chromatin within nuclei, Hoechst 33342 stain. (**C**) G4 structures within nuclei, BG4 antibody staining. (**D**) 1D3 antibody staining of hemin. (**E**) Anti-citrullinated histone H3 antibody staining of histone H3. (**F**) Merge of panels (C) and (D), showing possible colocalization of G4 structures and hemin. (**G**) Merge of panels (B)–(E) portraying defined cellular boundaries with intracellular labeling. (**H**–**N**) *Escherichia coli* stimulated neutrophils. (**H**) Bright field. (**I**) Extruded NETs DNA, Hoechst 33342 stain. (**J**) BG4 antibody staining of G4 structures within NETs. (**K**) 1D3 antibody staining of hemin with NETs. (**L**) Anti-citrullinated histone H3 antibody staining of histone H3 within NETs confirming the formation of NETs after *E. coli* stimulation. (**M**) Merge of panels (J) and (K), showing the possible colocalization of G4 structures and hemin. (**N**) Merge of panels (H)–(N) showing potentially extensive colocalization of G4 and hemin on NETs, evidenced by the Manders’ coefficient of 0.998 for G4 overlapping hemin and 0.843 for hemin overlapping G4. (**O**) Intact cell numbers under microscopy field of view. (**P**–**S**) Fluorescent area % under field of view. Scale bar = 25 μm. **** indicates *P*-value < 0.0001.

To determine if G4 and hemin are endogenously present within neutrophils and extruded NETs, we performed *in vitro* immunofluorescence co-localization of G4 and hemin on protein-bound crude NETs. NETosis was stimulated using *E. coli* in negatively selected neutrophils from healthy donors. NETs formation was confirmed by labeling with anti-citrullinated histone antibody and subsequently examined using bright field and fluorescent microscopy (Figure 1). Hemin and G4 were subsequently labeled with the highly specific anti-hemin monoclonal antibody and BG4 antibody, respectively, after confirming its specificity (Supplementary Figure S1). Intact neutrophils were observed prior to stimulation with *E. coli* with clearly defined boundaries for the DNA and G4 staining (Figure 1A–G). As expected, we observed minimal signal from the anti-citrullinated histone or anti-myeloperoxidase (Supplementary Figure S2) antibody in unstimulated neutrophils confirming that the isolated neutrophils were native and unstimulated. Stimulation with *E. coli* resulted in NETs as observed by the lack of defined cellular boundaries, formation of mesh-like structure in the DNA and G4 staining, strong signal from the citrullinated histones and myeloperoxidase (Figure 1H–N, and Supplementary Figure S2H–N) and the significant increase in the fluorescent signal area in the images captured. Additionally, estimating the Manders’ coefficient of overlap after performing automatic Costes’s thresholding of Figure 1J and K showed that 99.8% of the G4 signal overlapped with hemin while 84.3% of the hemin signal overlapped with G4, indicating the presence of stable G4 structures in the extruded DNA of NETs and suggesting a considerable degree of putative colocalization between G4 structures and hemin on the NETs. Similar observations were made in the extruded DNA of NETs induced by PMA and *P. aeruginosa* lipopolysaccharide (Supplementary Figure S3).

### G4/H in NETs possess DNAzyme peroxidase activity

**Figure 2.**
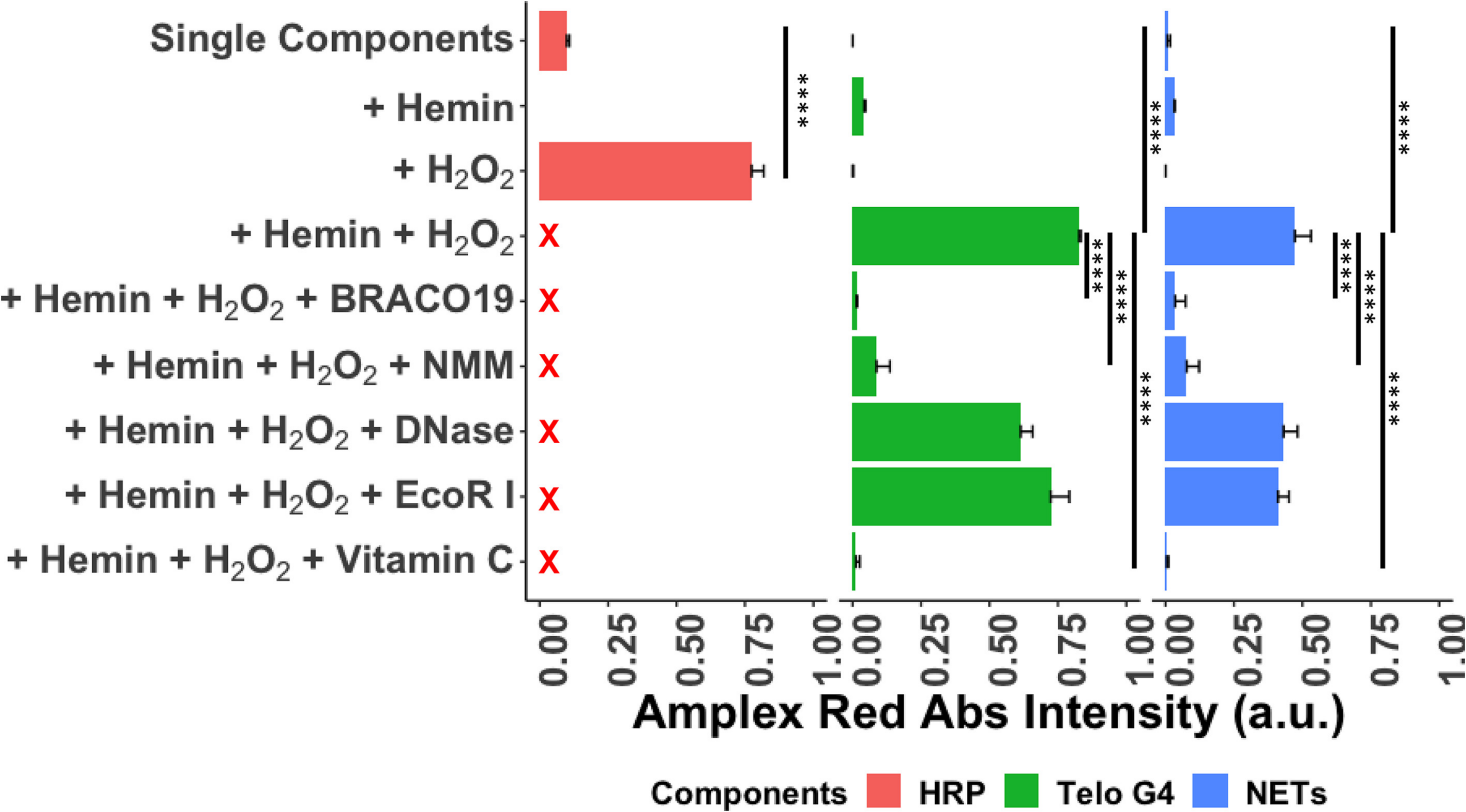
Peroxidase activity of G4/H DNAzyme in purified NETs DNA using AR. Peroxidase activity using equal masses of Telo G4 and purified NETs DNA were assayed with various combinations of individual components, G4 specific binding inhibitors (BRACO19 and NMM) and antioxidant (vitamin C). Telo G4 + hemin + H_2_O_2_ and HRP + H_2_O_2_ used have comparable activity. NETs + hemin + H_2_O_2_ had greater activity than individual components and 7.6x higher than hemin alone. Peroxidase activity was abrogated by G4 inhibitors and antioxidant. All experiments were performed in triplicate with mean and standard deviation plotted. One-way ANOVA test with Tukey’s honestly significant difference test performed to compare the different combinations. **** indicates *P*-value < 0.0001.

We next sought to determine if the G4/H complexes in NETs possessed the same DNAzyme peroxidase activity to produce FRs. Isolated neutrophils from a healthy donor were stimulated with PMA and the NETs DNA was then purified. We assessed the DNAzyme activity of purified NETs DNA to ensure we were only assaying the G4/H activity, excluding NETs-associated proteins. To compare the activity against that of HRP and commercial synthetic telomeric G4 complexed with hemin (Telo G4/H), we generated a standard curve of HRP activity on AR and determined the amount that 100 ng of Telo G4/H possessed the same activity as 0.66 mU of HRP (Supplementary Figure S4). For all subsequent experiments, we used the same mass of Telo G4 and purified NETs DNA. The peroxidase activity of the individual G4 and hemin components was assessed, revealing no significant observable activity. However, in the presence of H_2_O_2_, when hemin was combined with NETs G4, the resulting G4/H complex exhibited 7.6 times higher peroxidase activity compared with hemin alone. Purified NETs DNA, on average, demonstrated only 57% enzymatic activity compared with Telo G4/H despite the equal masses. This observation can be attributed to the likely lower abundance of G4 structures within the purified NETs DNA compared with the Telo G4 or potential destabilization of the G4 structures during genomic DNA purification. Notable results were obtained when investigating the impact of various treatments on the DNAzyme activity. The G4-specific competitive inhibitor BRACO19, the G4-binding hemin analog NMM and the FR scavenger vitamin C were found to abrogate the NETs DNAzyme activity by 92.5%, 83.5% and 99%, respectively. In contrast, surprisingly, treatment with nucleases, including DNase I or EcoR I, did not result in a discernible reduction in enzymatic activity (Figure 2).

### G4/H DNAzyme in NETs produces FR and exerts a localized, concentrated effect on trapped bacterial cells

**Figure 3.**
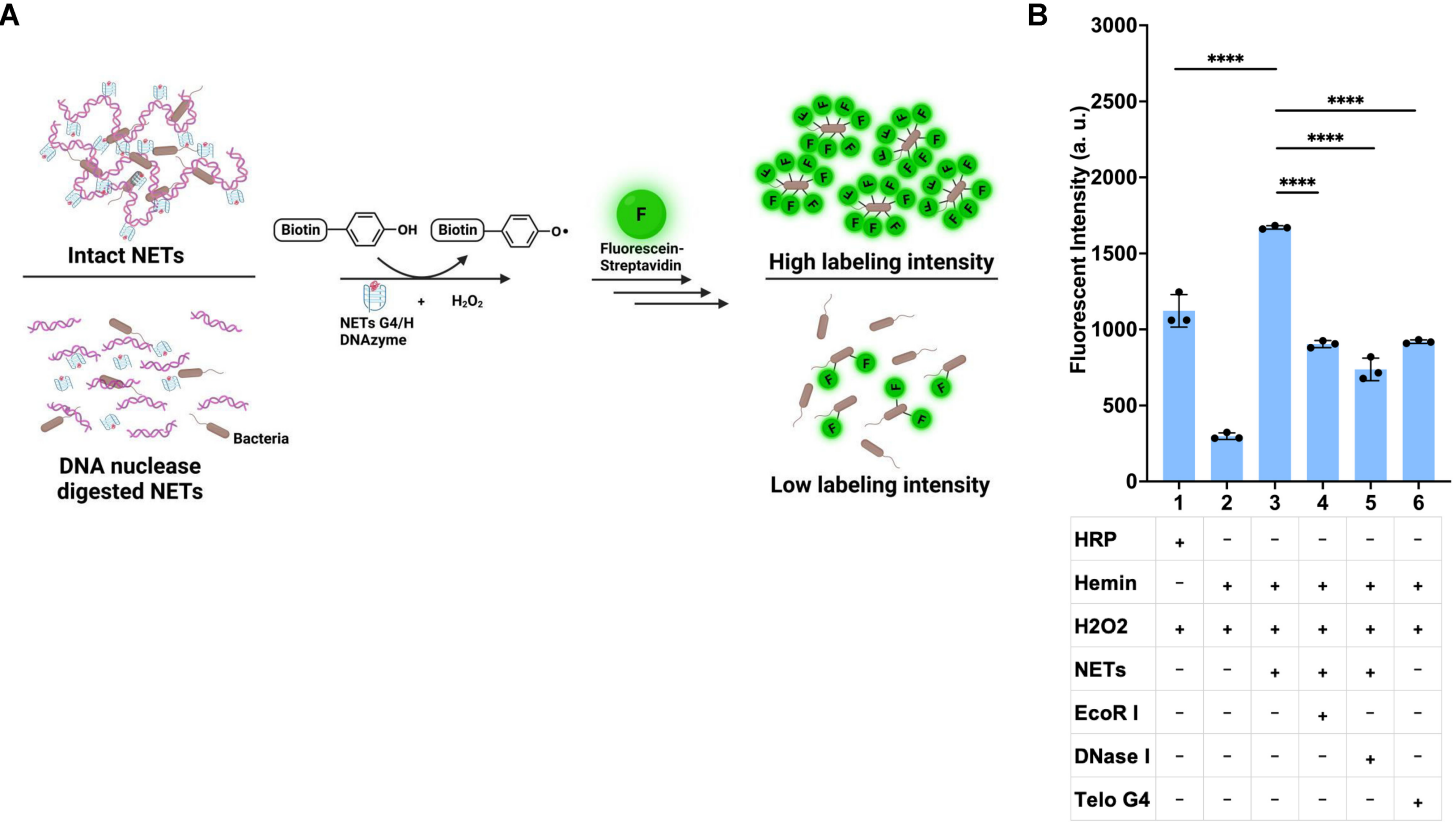
NETs DNA-based proximity labeling assay. (**A**) Schematic of proximity labeling assay. Labeling of bacteria trapped in purified NETs DNA, versus untrapped bacteria in nuclease-digested NETs DNA, with biotin-phenoxyl radicals by the DNAzyme activity of G4/H complexes. (**B**) Purified NETs DNA from PMA-stimulated neutrophils incubated with EC, biotin-phenol, hemin, H_2_O_2_ and fluorescein–streptavidin to allow for bacterial surface labeling. Upon digestion of NETs DNA with EcoR I and DNAse I on the same bacteria, the resulting fluorescent intensity showed a reduced labeling effect comparable to that of free-floating Telo G4 (∼54%) compared with intact NETs. (*n* = 3 biologically independent experiments; bars represent mean signal, and error bars denote s.e.m.; one-way ANOVA performed; **** indicates *P*-value < 0.0001).

In order to ascertain the type of FR generated by the G4/H DNAzyme in NETs, their potential impact on bacteria and the role of NETs DNA structure, we performed the proximity labeling assay on EC. Proximity labeling exhibits optimal effectiveness within the 1–10 nm radius (30). We used biotin-phenoxyl radical, resulting from the generation of hydroxyl radicals (OH•) by the G4/H DNAzyme (31,32), to fluorescently label the surface of bacteria trapped within purified NETs DNA. The generated biotin-phenoxyl radical selectively reacts with electron-rich amino acids, such as tyrosine, tryptophan and others, located in close proximity on the bacterial surfaces. This reaction leads to fluorescence labeling upon treatment with fluorescein–streptavidin. EC was incubated with a biotin-phenol substrate, fluorescein–streptavidin, and a combination of purified NETs DNA from PMA-stimulated neutrophils with NETs DNA of fragment size over 10 kilobases (Supplementary Figure S5) (33), hemin, and H_2_O_2_. Baseline fluorescence intensities from individual components were minimal. While the proximity labeling assay confirmed the production of OH• by the G4/H DNAzyme in NETs, the amount of labeling by OH• produced by the G4/H DNAzyme when the NETs DNA structure was maintained was 137% and 160% greater than that by HRP and free-floating Telo G4, respectively (Figure 3), despite being normalized according to activity in the standard curve described above suggesting an increased effect because of the structure of intact NETs. This was confirmed by digesting the NETs DNA structure with EcoR I or DNase I, both of which reduced the labeling to 44% and 54%, respectively, comparable to the Telo G4/H, likely because even short oligonucleotides can result in DNA–protein interactions on the cell surface (34,35). These findings support the local concentrated effects of the G4/H DNAzyme on trapped bacteria in NETs, especially since mass for mass, we observed earlier that Telo G4/H has greater peroxidase activity than purified NETs DNA G4/H DNAzyme. As expected, the trapping effect was not readily apparent for SA (Supplementary Figure S6) since it is known to evade NETs through its ability to release nucleases to breakdown DNA (36) resulting in comparable signal between free-floating Telo G4, intact NETs and additional EcoR I or DNase I treatments.

### G4/H DNAzyme is physiologically active and the major contributor of bactericidal activity in NETs

**Figure 4.**
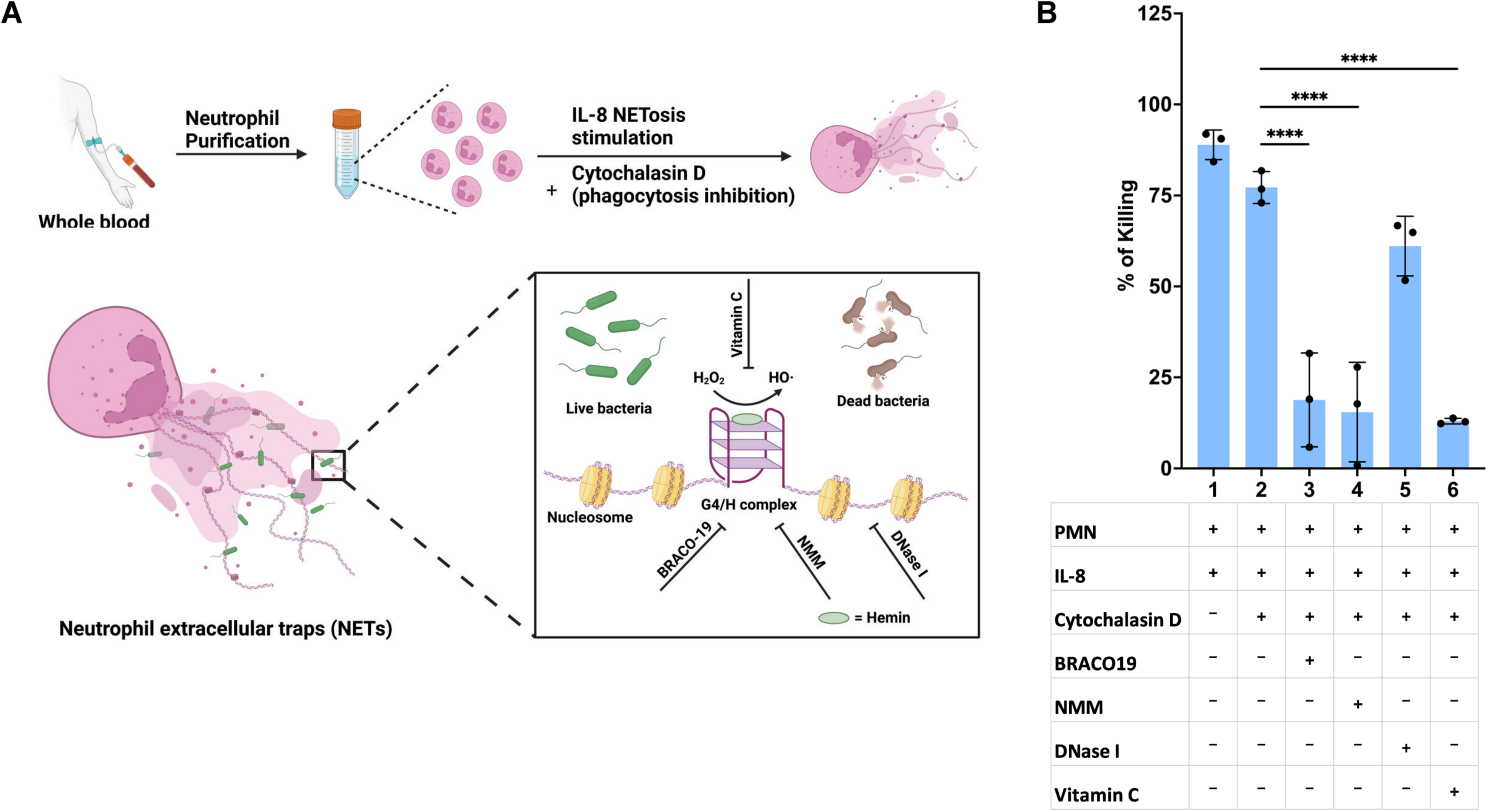
*Ex vivo* bactericidal activity of the G4/H DNAzyme against EC. (**A**) Schematic of the *ex vivo* bactericidal assay. Neutrophils from whole blood were stimulated with IL-8 and treated with various combinations of antiphagocytic cytochalasin D, G4 specific inhibitor BRACO19, G4 binding hemin analog NMM and DNase I with no additional hemin or H_2_O_2_. (**B**) Bactericidal activity of isolated neutrophils through NETs against EC. Approximately 80% of inoculated EC was killed by IL-8-stimulated NETs. Phagocytosis accounts for an additional ∼10% of killing. Abrogation of NETs killing by G4-specific inhibitors like BRACO19, NMM or antioxidant vitamin C (<20%). DNase I treatment only reduced killing by ∼20%. (*n* = 3 biologically independent experiments; bars represent mean signal, and error bars denote s.e.m.; one-way ANOVA performed; **** indicates *P*-value < 0.0001).

We next sought to determine if the G4/H DNAzyme was physiologically active and measure its contribution to the bactericidal activity of NETs *ex vivo*. We hypothesized that all essential components for its activity were naturally present in blood, for example during inflammation, hemolysis increases extracellular hemin levels, and dissolved H_2_O_2_ in plasma can be as high as 50 μM (37,38). To demonstrate this phenomenon, we conducted an *ex vivo* experiment with neutrophils isolated from a healthy individual against EC, following a previously published protocol with minor modifications (1). EC was incubated with neutrophils in DPBS containing heat-inactivated human serum along with various combinations of the physiologically relevant chemokine and activator, IL-8, cytochalasin D (an antiphagocytic agent), BRACO19, NMM and DNase I with no additional hemin or H_2_O_2_. Stimulation of neutrophils with IL-8 led to approximately 85% eradication of EC (Figure 4) despite the ability of EC to encode catalase to protect it from H_2_O_2_ toxicity. Interestingly, inhibiting phagocytosis had only a marginal impact on the bactericidal efficacy, indicating that the majority of the observed killing in our assay can be attributed to NETs. Administration of NMM and BRACO19, which specifically target the G4/H structure of NETs, resulted in a substantial reduction of the bactericidal effect to <20%, ∼74% reduction in killing. Interestingly, quenching FR through treatment with vitamin C resulted in <20% bactericidal effect similar to treatment with BRACO19 and NMM suggesting that in our *ex vivo* model, the other mechanisms of bacterial killing in NETs contributed minimally and the majority of the FR was generated by the G4/H DNAzyme. Similar results were observed with SA (Supplementary Figure S8), however, with less overall killing than with EC likely because it encodes extracellular nucleases. Remarkably, treatment with DNase I exhibited only a modest decrease of ∼20% in the bactericidal activity against EC but ∼80% decrease in killing of SA.

## Discussion

In this study, we assess the existence and functionality of G4s in the extruded DNA of human NETs. G4 structures in the genomic landscape of humans have been detected to sequester free hemin both by DNA and RNA forms to protect the cell from oxidative damage and affect transcriptional control (16,18). However, these studies did not describe the peroxidase activity of the biological G4/H complex in humans, while such peroxidase activity has been demonstrated in Drosophila embryonic salivary glands (39). In the matrices of biofilms formed by bacteria like *P. aeruginosa* and *Staphylococcus epidermidis*, G4s were found to be abundant with potential roles in forming the gel like structure in biofilms (40,41) but nothing is known about the extracellular DNA of NETs. Immunofluorescence staining of NETs induced by PMA, *P. aeruginosa* lipopolysaccharide and *E. coli* with G4 specific antibody, BG4, all showed strong labeling in confirmed NETs (Figure 1, and Supplementary Figures S2 and S3) suggesting stable G4s in extruded human DNA. While chromatin accessibility in neutrophils vary in response to different stimuli (42), our data suggests that upon decondensation and extrusion, the exposed DNA carries potentially >700 000 G4s. The immunofluorescence staining also revealed potential extensive colocalization between the G4s and hemin in the extruded DNA (Figure 1) and while intracellular G4/H interactions (16–18,39) have previously been described in other cell types, this co-localization offers evidence for the existence of extracellular G4/H in a biological setting.

Hemin is an important co-factor that is typically believed to be generated from the turnover of old red blood cells, and the extracellular levels of hemin can be exacerbated by hemolysis during infection. Surprisingly, we observed a strong hemin signal in both neutrophils from healthy donor and their extruded NETs without the addition of extraneous hemin in our experiment. Given that G4s in the genome are believed to sequester free hemin to protect from hemin-derived oxidative stress (17,18), it is possible that the hemin was sequestered by the G4s in neutrophils prior to the blood draw for the assay. However, since extracellular hemin can induce NETosis of neutrophils in a dose-dependent manner (43–45), it reduces the likelihood of the neutrophils in this study having come across free hemin prior to blood draw and raises the possibility of endogenously produced hemin in neutrophils.

Synthetically generated complexes of G4/H DNAzymes have been studied for years with various applications in biosensors and other biochemical assays (21,46,47), but their physiological existence and biological functions are not widely understood. Here, we demonstrate the existence and physiologically relevant biological activity of the G4/H DNAzyme in the extruded, decondensed chromatin present in NETs. While the imaging based colocalization of G4/H provided a qualitative measure of the proximity between the two on NETs, we assessed their activity when combined *in vitro*. The G4/H DNAzyme on NETs mimics peroxidase activity in producing OH• from H_2_O_2_ and has stronger activity than hemin alone (Figures 2 and S4), supporting findings from previous studies (19,21,22,32,48). However, the DNAzyme exhibited overall lower activity compared with HRP, which is supported by recent investigations that revealed a dependency on the specific substrate being catalyzed (39,49). Interestingly, despite their high specificity for binding to G4, we observed negligible remnant DNAzyme activity even after blocking with BRACO19 and NMM, which however, could be a result of hemin binding to the duplex DNA to mimic weak peroxidases (50,51). Additionally, treatment with nucleases, including DNase I or EcoR I, did not result in a discernible reduction in enzymatic activity suggesting that these nucleases were unable to degrade the non-canonical G4 structures in NETs as was previously shown in bacterial biofilms (41,52). The nuclease treatment, however, did reduce the proximity labeling by >40% compared with when the NETs were intact (Figures 3 and S6). While monomeric G4/H DNAzyme units have lower activity than peroxidase enzymes like HRP, it is possible that G4/H DNAzyme in bacteria-bound NETs formed multimeric units that have increased synergistic activity (53,54) and with potentially >700 000 G4/H localized on NETs, greater quantities of FR with higher local concentrations could be produced.

FR are bactericidal and OH• is the most reactive FR *in vivo* (55) and while NETs are known to produce various FR like HOCl and NO• through the action of myeloperoxidase and nitric oxide synthase respectively, no enzymatic generators of OH• in NETs were previously known. NETs mediated killing has largely been attributed to associated proteins and granules and more recently, the largest component of NETs, its DNA, was determined to have potential for bactericidal activity through chelating properties of the DNA backbone (1,12). Our *in vitro* and *ex vivo* assays highlight a potent role for the G4, a non-canonical DNA secondary structure of NETs, as a major contributor in clearing pathogens (Figure 4, and Supplementary Figures S7 and S8). Our evidence suggests that not a protein enzyme but the G4/H DNAzyme produces OH• in NETs that is actively bactericidal. In fact, the potency of this mechanism is so strong that EC were killed by the DNAzyme despite the bacterium's ability to encode the catalase enzyme that degrades H_2_O_2_ to water and oxygen, likely because synthetic DNAzymes have been shown to have a higher affinity for H_2_O_2_ than catalases (56,57). While we observed strong killing of both EC and SA by the DNAzyme, evidenced by only ∼25% killing by NETs associated proteins and granules when G4 was blocked, DNase I mediated disruption of NETs showcased mixed results. Although an integral aspect of NETs experiments over the decades, the heterogeneity we observed in the extent of killing upon DNase I treatment is supported by previous studies. While we see only a 20% reduction in overall killing of EC when blocked with DNase I, we do observe a significant difference in the killing of SA (Fig S8). The results from SA mimic findings from previous studies (1,12). One possibility is that a species dependent response to the NETs killing mechanisms is in play dependent on the need for effective trapping of the bacterium. Especially since prior work has shown that bacterial species differ in their susceptibility to extracellular DNA, in a time dependent manner (12), and certain species like *Streptococcus pneumoniae* are resistant to extracellular DNA based killing (58). Further, in line with our data supporting species specific response to NETs based killing, prior studies have shown that DNase treatment of NETs reverses killing of SA, *E. coli* and *P. aeruginosa* (12) but not in *S. flexneri* (58) and an enterovirulent diffusely adherent strain of *E. coli* (59).

Trapping of bacteria in NETs DNA subjects it to increased local concentrations of the OH• resulting in the mechanism being the driver of bactericidal activity in NETs. While our demonstration is in an extracellular setting, our findings raise the question of the effect of highly localized OH• on the genome intracellularly, especially when hemin is sequestered. It is possible that one of the hundreds of putative G4 interacting proteins (60) recently discovered dampens the DNAzyme activity within the cell, but further work is needed to elucidate these mechanisms. Additionally, we only assayed the role of the G4/H generated FR in bactericidal activity associated with NETs while there may be other biological consequences. For example, FRs from NETs have been associated with host tissue damage and the formation of posttranslational modifications on NETs-associated histones resulting in autoimmune disorders like systemic lupus erythematosus (61–65). We offer a paradigm shift in the current understanding of the role of the NETs DNA – beyond a scaffold to the driver of potentially multiple biological consequences of NETs.

## Supplementary Material

gkae1262_Supplemental_File

## Data Availability

All data supporting the findings of this study are available within the article and its supplementary material.
