# Supplementary material for: Neutrophil extracellular traps have active DNAzymes that promote bactericidal activity
Source: Nucleic Acids Res. 2024 Dec 31;53(3):gkae1262. doi: 10.1093/nar/gkae1262 (PMC11797030; doi:10.1093/nar/gkae1262)
Supplement: gkae1262_Supplemental_File [file gkae1262_supplemental_file.docx]

Supplementary Materials for

Neutrophil Extracellular Traps have active DNAzymes that promote bactericidal activity

Ti-Hsuan Ku *et al.*

*Corresponding author. Email: syang5@stanford.edu

**This PDF file includes:**

Figs. S1 to S8


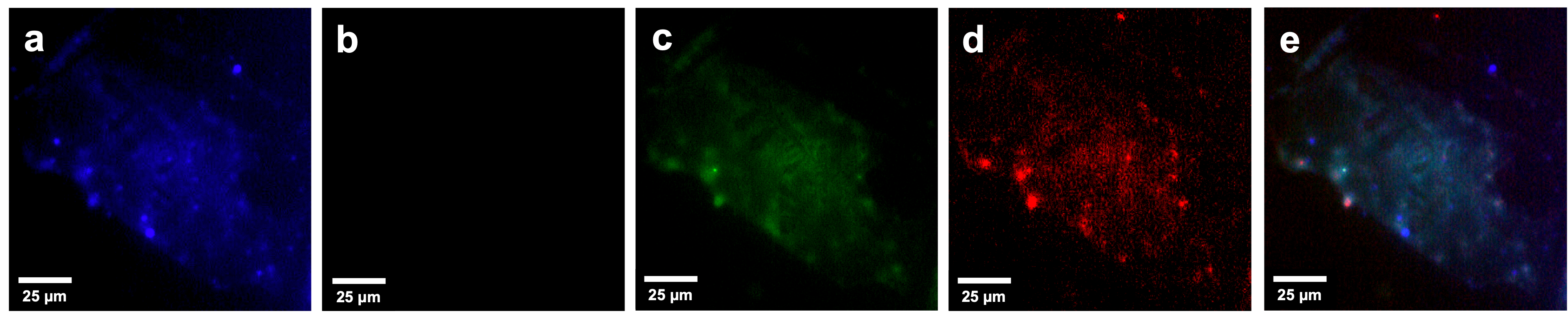


**Fig. S1. BG4 scFv antibody negative control.** (a) Extruded NETs DNA, Hoechst 33342 stain. (b) Lacking of BG4 antibody staining of G4 structures within NETs. (c) 1D3 antibody staining of hemin within NETs. (d) Anti-Citrullinated Histone H3 antibody staining of Histone H3 within NETs. (e) Merge of a-d showing hemin and no G4 on NETs DNA demonstrating specificity of the BG4 antibody. Scale bar = 25 μm.


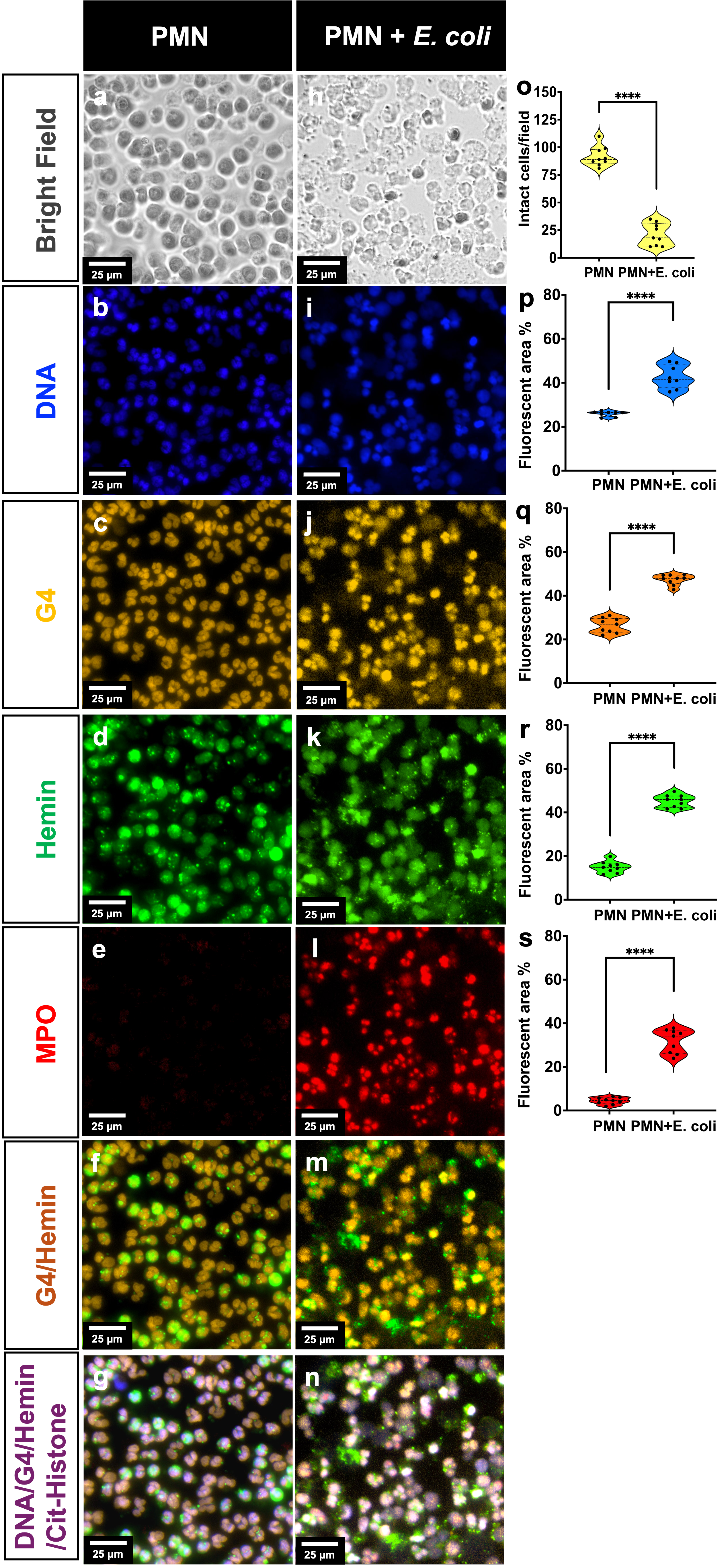


**Fig. S2. Immunofluorescence stain of G4/H colocalization on NETs.** (a-g) Unstimulated neutrophils (PMN). (a) Bright field. (b) Condensed chromatin within nuclei, Hoechst 33342 stain. (c) G4 structures within nuclei, BG4 antibody staining. (d) 1D3 antibody staining of hemin. (e) Anti-Citrullinated Histone H3 antibody staining of Histone H3. (f) Merge of c and d, showing the colocalization of G4 structures and hemin. (g) Merge of b-e portraying defined cellular boundaries with intracellular labeling. (h-n) *E. coli* stimulated neutrophils. (h) Bright field. (i) Extruded NETs DNA, Hoechst 33342 stain. (j) BG4 antibody staining of G4 structures within NETs. (k) 1D3 antibody staining of hemin with NETs. (l) Anti-MPO antibody staining of MPO within NETs confirming the formation of NETs after *E. coli* stimulation. (m) Merge of j and k, showing the colocalization of G4 structures and hemin. (n) Merge of h-n showing extensive colocalization of G4 and hemin on NETs. (o) Intact cell numbers under microscopy field of view. (p-s) Fluorescent area % under field of view. Scale bar = 25 μm. **** indicates p-value < 0.0001.


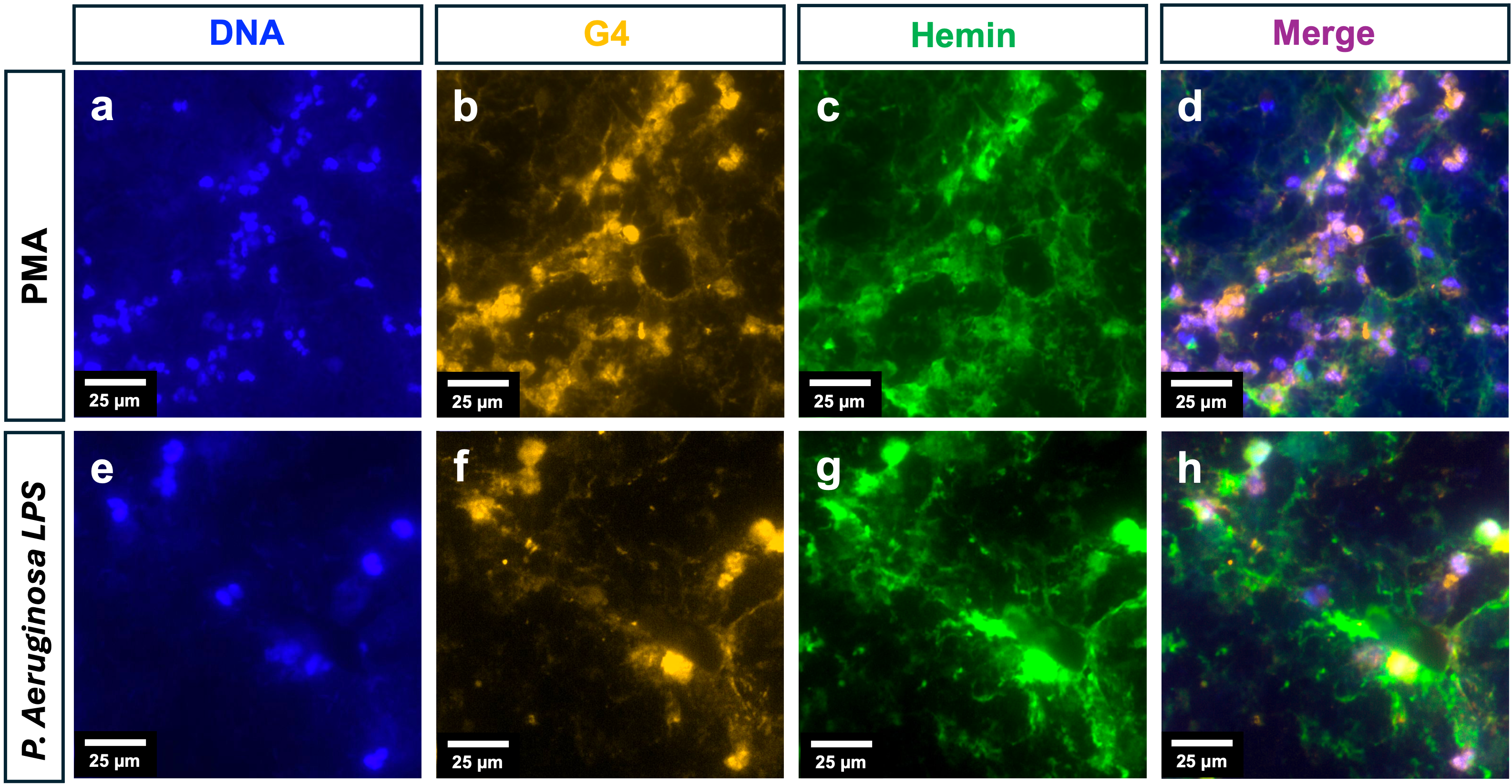


**Fig S3. PMA-stimulated and *P. aeruginosa* lipopolysaccharide-stimulated NETosis.**(a) Extruded NETs DNA, Hoechst 33342 stain. (b) BG4 antibody staining of G4 structures within NETs. (c) 1D3 antibody staining of hemin with NETs. (d) Merge of a-c showing extensive colocalization of G4 and hemin on NETs. (e) Extruded NETs DNA, Hoechst 33342 stain. (f) BG4 antibody staining of G4 structures within NETs. (g) 1D3 antibody staining of hemin with NETs. (h) Merge of e-g showing extensive colocalization of G4 and hemin on NETs. Sacle bar = 25 μm.


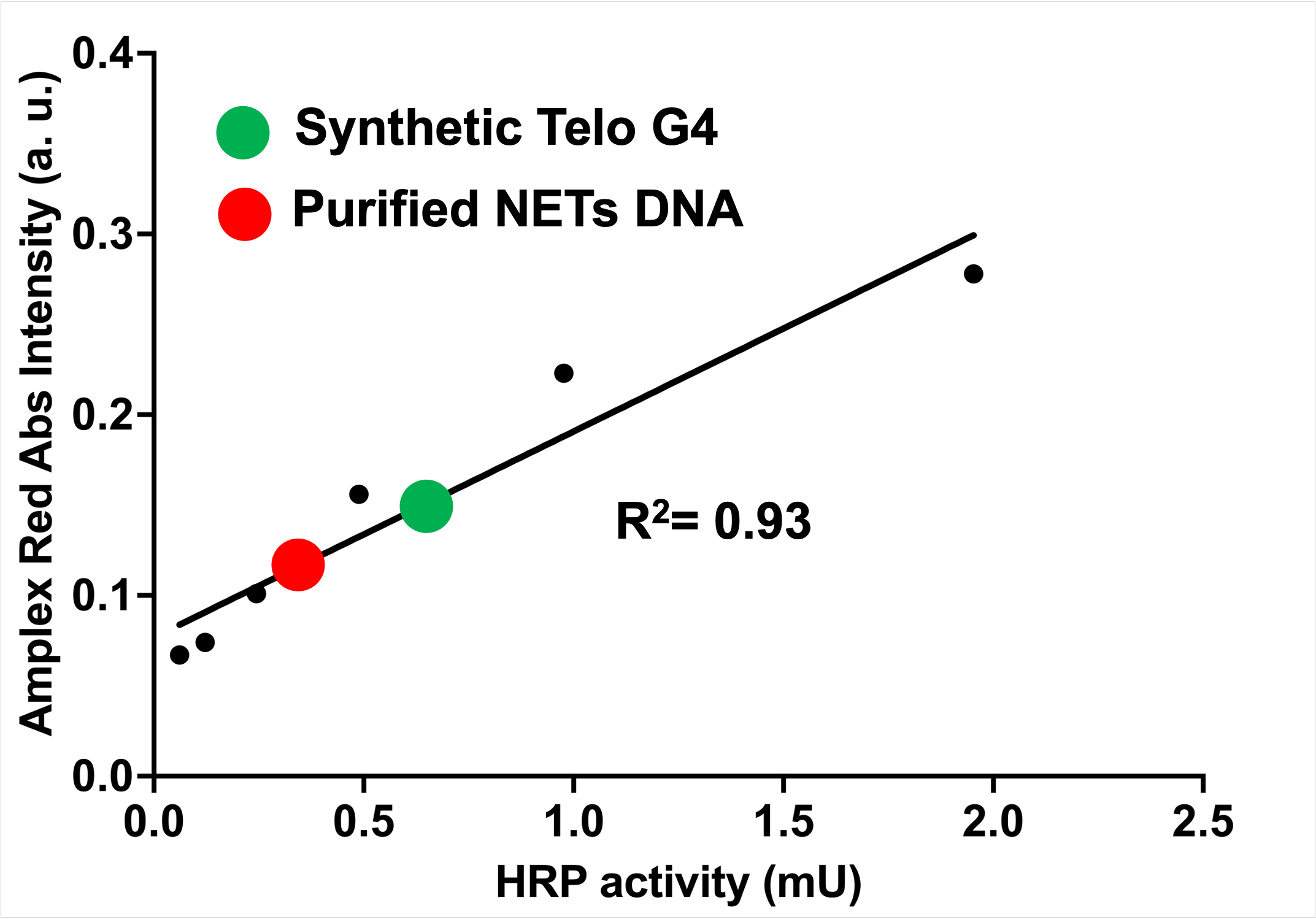


**Fig. S4. Estimating amount of Telo G4 needed to achieve same HRP activity.** HRP standard curve was stablished by using Amplex UltraRed as substrate. 100 ng of NETs DNA and Telo G4 showed similar activity to 0.3 mU and 0.66 mU of HRP respectively.


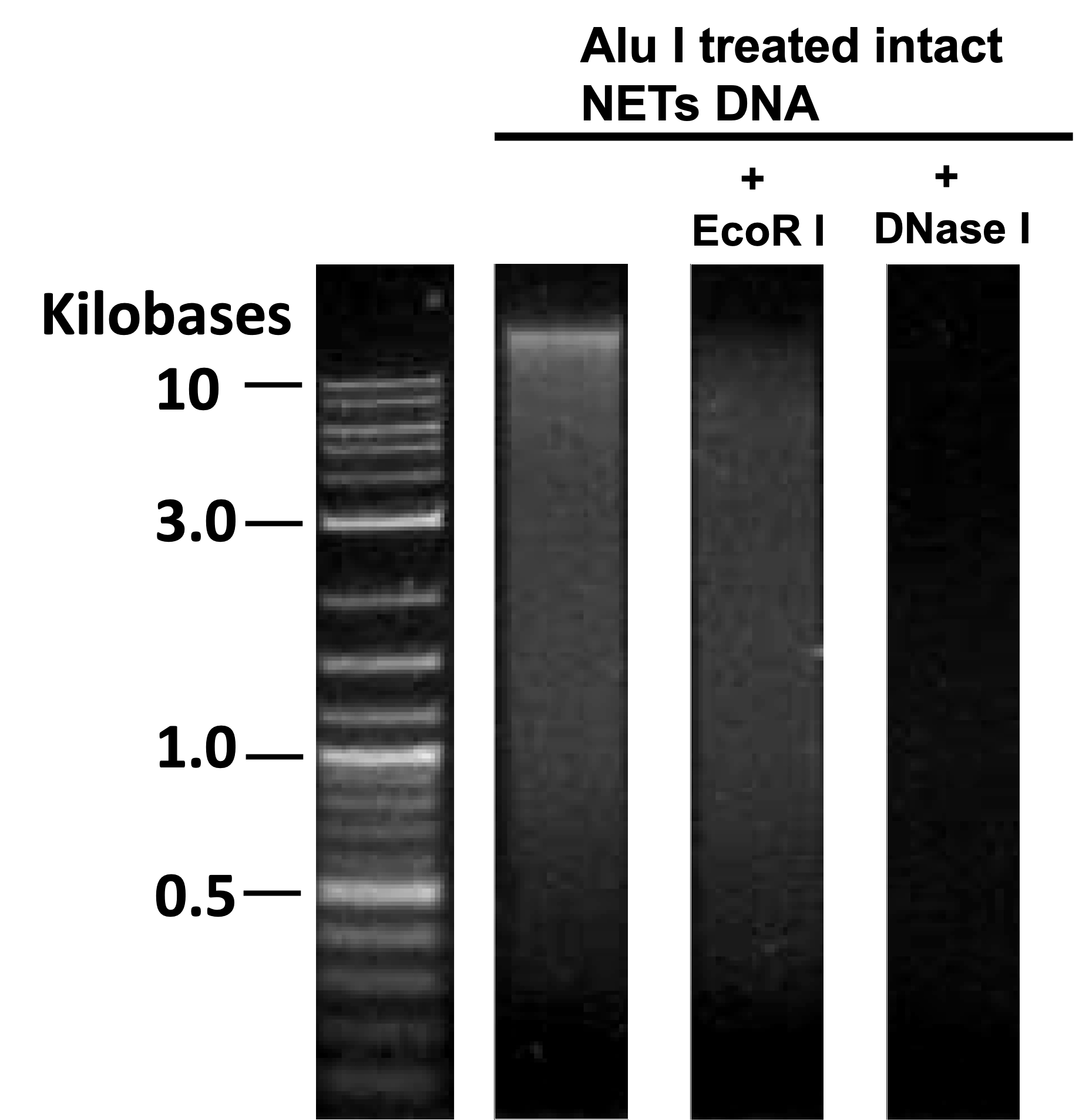


**Fig. S5 Purified NETs DNA fragmentation.** Purified NETs DNA gel electrophoresis after Alu I digestion and genomic DNA purification with or without further EcoR I, DNase I treatment. DNA migration took place in 1.3% agarose gel staining with ethidium bromide. The >10 Kb band indicates the integrity of NETs DNA after the purification and collection process.


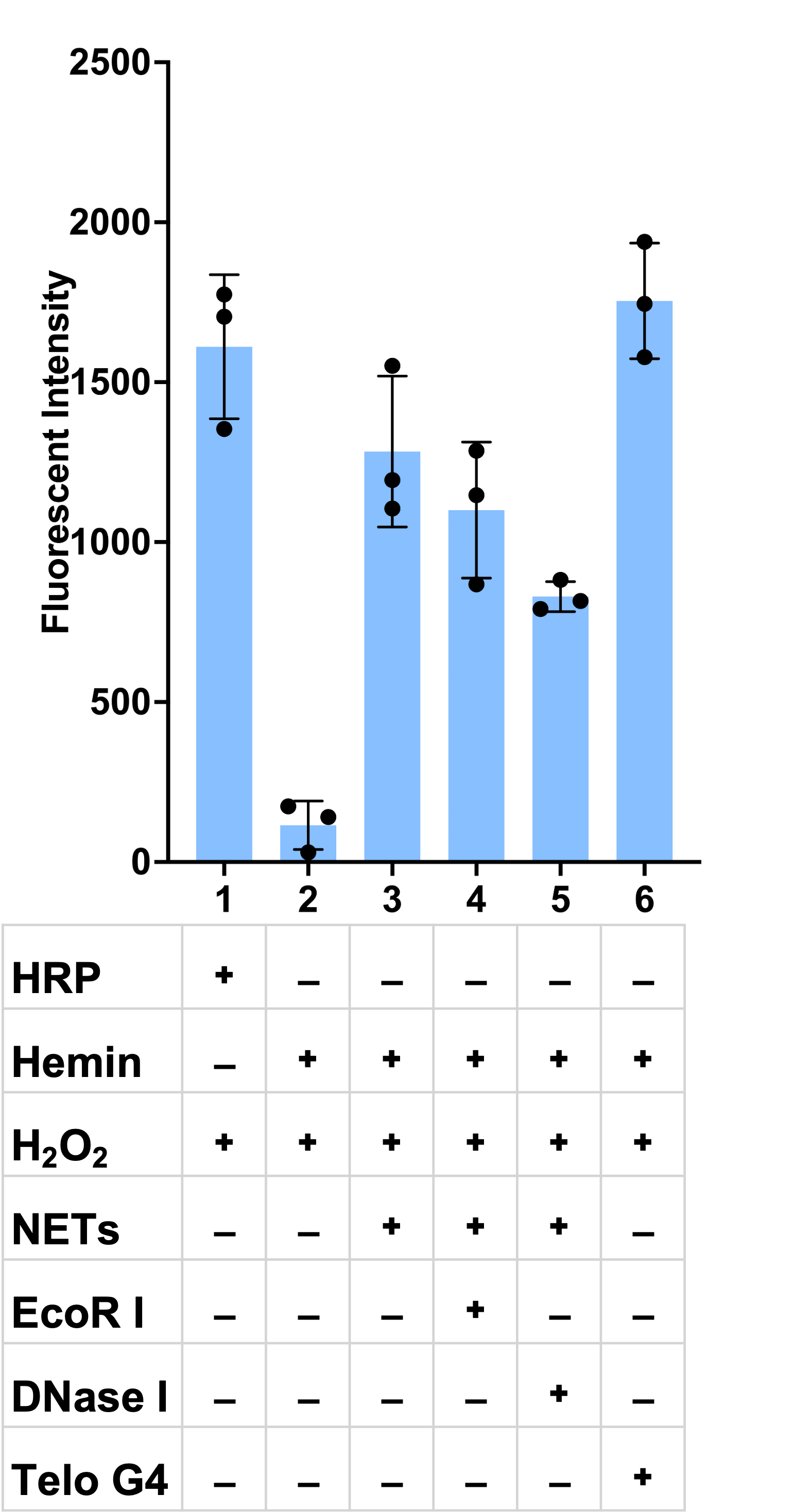


**Fig. S6 Proximity labeling of *Staphylococcus aureus* (SA).** Purified NETs DNA from PMA-stimulated neutrophils incubated with SA, biotin-phenol, hemin, H_2_O_2_, and fluorescein-streptavidin to allow for bacterial surface labeling. Upon digestion of NETs with EcoR I and DNAse I on the same bacteria, the resulting fluorescent intensity showed a reduced labeling effect compared to intact NETs. (n = 3 biologically independent experiments; bars represent mean signal, and error bars denote s.e.m. one-way ANOVA performed; no significant difference was found between HRP, NETs, Telo G4, DNase and EcoR I treatment.)


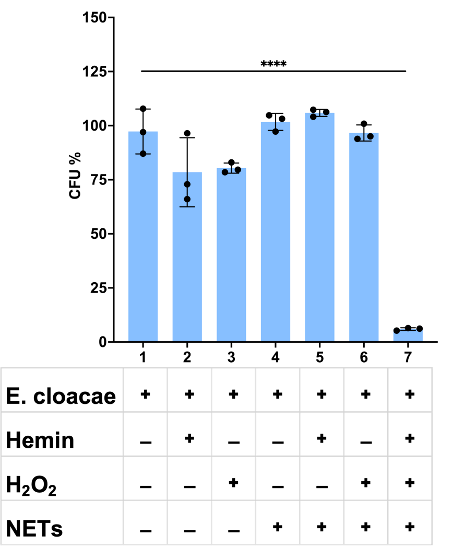


**Fig. S7 *In vitro* bactericidal assay.** EC was incubated with various combinations of purified NETs DNA from PMA-stimulated neutrophils, hemin, and H_2_O_2_ to allow for bacterial killing. Plate colony counting was performed after overnight incubation. Individual components were unable to kill EC. Only complete DNAzyme with H_2_O_2_ killed EC (n = 3 biologically independent experiments; bars represent mean signal, and error bars denote s.e.m. one-way ANOVA performed; **** indicates p-value < 0.0001)


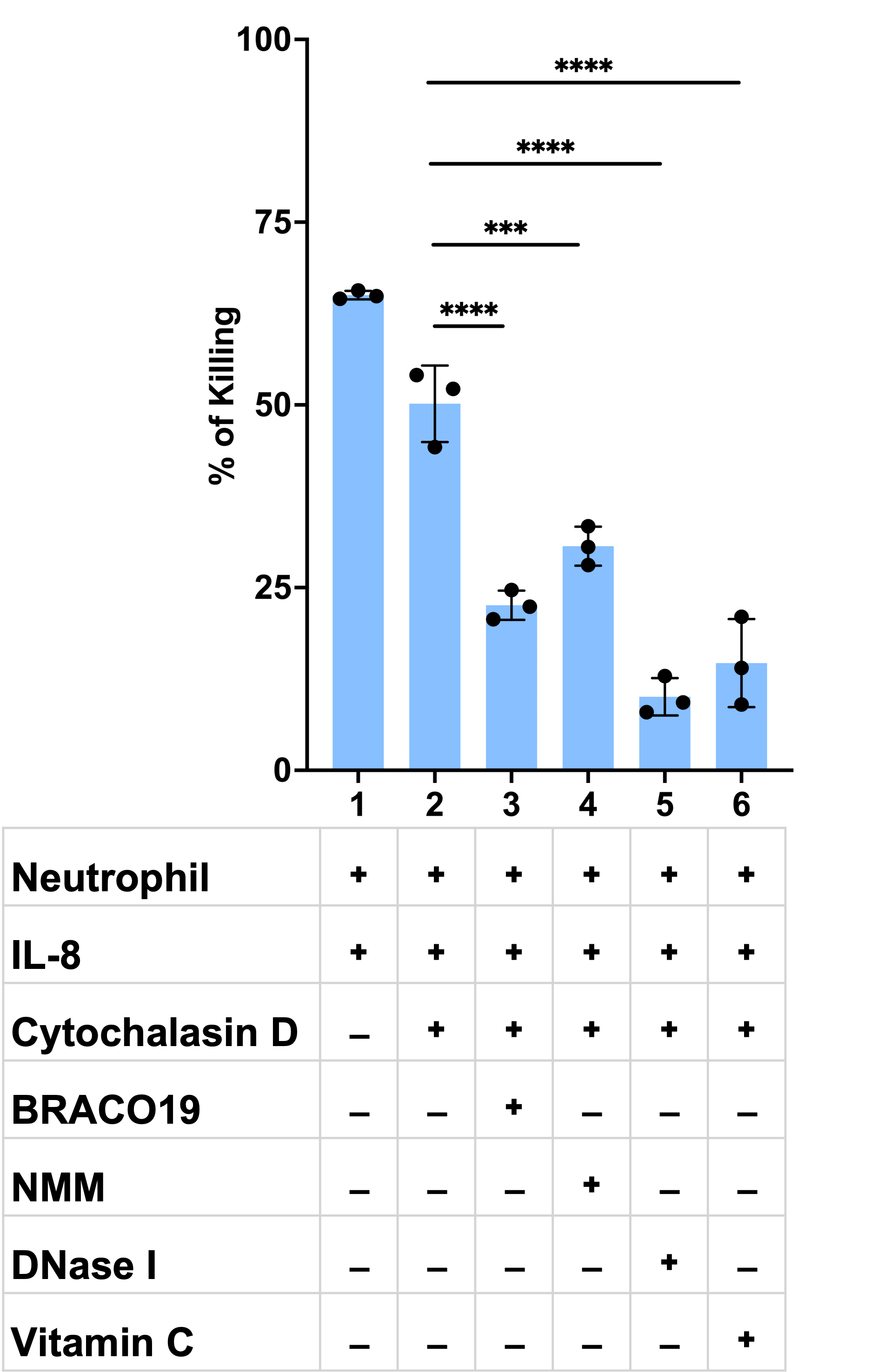


**Fig. S8 *Ex vivo* bactericidal activity of the G4/H DNAzyme for *Staphylococcus aureus* (SA).** Bactericidal activity of isolated neutrophils through NETs. ~ 63% of inoculated SA was killed by IL-8-stimulated NETs. Phagocytosis account for an additional ~13% of killing. Abrogation of NETs killing by G4-specific inhibitors like BRACO19, NMM, or antioxidant Vitamin C (< 25%). (n = 3 biologically independent experiments; bars represent mean signal, and error bars denote s.e.m.; one-way ANOVA performed; **** indicates p-value < 0.0001, *** indicates p-value < 0.001)
